# Introducing an on-site Helicopter Emergency Medical Service (HEMS) physician at the Emergency Medical Communication Centre - implications for dispatch precision at a Norwegian HEMS base

**DOI:** 10.1186/s13049-025-01396-1

**Published:** 2025-05-07

**Authors:** Ole Erik Ulvin, Oddvar Uleberg, Andreas Asheim, Helge Haugland

**Affiliations:** 1 Department of Research and Development, Norwegian Air Ambulance Foundation, Oslo, Norway; 2 Department of Emergency Medicine and Prehospital Services, St. Olav University Hospital, Trondheim, Norway; 3 Department of Anaesthesia and Intensive Care Medicine, St. Olav Hospital, Trondheim, Norway; 4 Department of Circulation and Medical Imaging, Faculty of Medicine and Health Sciences, Norwegian University of Science and Technology (NTNU), Trondheim, Norway; 5 Center for Healthcare Improvement, St. Olav Hospital, Trondheim, Norway; 6 Department of Mathematical Sciences, Faculty of Information Technology and Electrical Engineering, Norwegian University of Science and Technology (NTNU), Trondheim, Norway

**Keywords:** Air ambulances, Emergency Medical Dispatch, Emergency Medical Service Communication Systems

## Abstract

**Background:**

Dispatch precision of Helicopter Emergency Medical Services (HEMS) is a key topic in prehospital research. In Norway, the combined role of the HEMS physician on-call and the Emergency Medical Communication Centre (EMCC) physician has been challenged. This study aimed to evaluate the impact on HEMS dispatch precision by transferring the medical decision-making from an on-call HEMS physician to an on-site HEMS physician in the EMCC.

**Methods:**

In this quasi-experimental study, a HEMS physician was on-site in Trondheim EMCC during defined working hours from February 1st through July 5th, 2024. When on-site, the decision to dispatch Trondheim HEMS was made by this EMCC physician. Primary outcome was unnecessary HEMS dispatches, i.e. missions where neither advanced treatment nor logistical contributions were provided following HEMS dispatch. Secondary outcomes were HEMS alarm and activation time, rejected HEMS missions and National Advisory Committee for Aeronautics (NACA)-scores of encountered HEMS patients. Outcomes were analysed by difference-in-differences analyses.

**Results:**

785 HEMS missions were included in the analyses. There was no significant difference in the risk of an unnecessary mission (percentage point risk difference [RD] 5.6, 95% confidence interval [CI] -7.4–18.6) or the proportion of patients with NACA scores of 4 or higher (RD -5.8, 95% CI -17.9–6.3) following the intervention.

**Conclusion:**

We found no evidence of increased HEMS dispatch precision, measured by the proportion of missions without medical or logistical contributions, when transferring the medical decision regarding HEMS dispatch from the HEMS physician on-call to an on-site EMCC physician in this study.

**Supplementary Information:**

The online version contains supplementary material available at 10.1186/s13049-025-01396-1.

## Background

As a limited and specialized prehospital resource, appropriate use of Helicopter Emergency Medical Services (HEMS) has gained increased attention in recent years [1–4]. An increasing number of emergency calls in Western countries during recent decades further challenges the capacity of both the Emergency Medical Communication Centres (EMCC) and prehospital resources [5, 6]. The growing demands on emergency medical services (EMS) necessitate efforts to enhance the dispatch of prehospital resources, including strengthened clinical decision-making in the EMCC [7, 8].

In Norway, national legislation requires every EMCC to have a consultant physician available at all times to assist the EMCC operator on demand; this is typically the anaesthesiologist on-call at the nearest HEMS base [9]. The combined role of the EMCC consultant physician (EP) and the HEMS physician on-call has been challenged in public reports, particularly the restricted availability of the EP during and after HEMS missions, and the EP not being physically present with access to the same tools and information as EMCC operators [9, 10]. Holding two positions simultaneously might also represent a potential for mixing of roles when assessing clinical and operational considerations related to a mission [10, 11].

Previous publications have described positive effects on dispatch precision of prehospital resources and decision-making processes by involving physicians with direct access to telemedical solutions in the EMCC [12–14]. Various studies have also investigated different HEMS dispatch models, including dispatch by HEMS crews compared to dispatch centres, and comparisons of non-clinical dispatch models with paramedic-led dispatch in the EMCC [15–19]. However, there is currently a lack of evidence regarding the effects on HEMS dispatch by introducing an on-site physician in the EMCC. This study aimed to evaluate the impact on HEMS dispatch precision by transferring the medical decision-making from an on-call HEMS physician to an on-site HEMS physician in the EMCC.

## Methods

### Study setting

In Norway, EMCC operators receive emergency calls, perform initial triage and provide first aid advice by phone supported by the Norwegian Index for Emergency Medical Assistance, while simultaneously dispatching resources and coordinating the medical response [20–22]. Depending on case severity, this might include dispatching one or more prehospital response units (ground ambulance or boat) and, if necessary, the local general practitioner on-call. Based on national guidelines for HEMS activation, operators may also request a HEMS crew that can reach patients either by helicopter or a rapid response car, as appropriate. If so, the regional HEMS coordinator tasks the most appropriate HEMS unit [23]. The final decision on whether to respond to a HEMS mission request relies on medical considerations by the HEMS physician on-call, as well as operational aspects like weather conditions judged by the pilot.

The 753 600 inhabitants (2024) of the region of Central Norway are dispersed over 56 559 square kilometres [24]. St. Olav`s University Hospital in Trondheim is the tertiary referral centre amongst a total of eight hospitals in the region. The regional EMCC is located in Trondheim and staffed by six EMCC operators at daytime, five in the evenings and four at night on weekdays. All EMCC operators in Norway are healthcare professionals (e.g. emergency medical technicians, paramedics or nurses) [21]. In 2023, approximately 45 000 calls to the national emergency number 113 were received by Trondheim EMCC.

Trondheim HEMS base operates an Airbus Helicopter H145 and a rapid response car staffed with a HEMS crew member (HCM), a pilot and an anaesthesiologist. The eight physicians on the base are experienced in prehospital emergency care with a minimum of 10 years working in this HEMS service. In addition to responding to HEMS mission requests, the HEMS physician on-call also gives medical advice to Trondheim EMCC as its EP. This consulting is primarily on medical treatment and prehospital logistics in missions where HEMS is not dispatched.

### The intervention

From February 1st through July 5th, 2024, an on-site EP was present in Trondheim EMCC at daytime during weekdays (Monday to Thursday from 10am to 10 pm, and Fridays from 08am to 4pm) except for public and school holidays. These intervention times were chosen for reasons of feasibility and to cover the periods of high HEMS activity. Seven out of eight eligible HEMS physicians from the Trondheim HEMS base agreed to participate as on-site EP, in addition to their ordinary HEMS shifts during the intervention period. Outside working hours with an on-site EP, the HEMS physician on-call functioned both as EP and HEMS physician according to usual practice.

Prior to the intervention, the participating physicians were trained in standard EMCC operations and procedures, including using the AMIS software (EMCC database; CSAM Health AS, Oslo, Norway) and the Integrated Communication and Control System (ICCS; Frequentis AG, Vienna, Austria) [25]. The on-site EP was located at a dedicated desk in the EMCC, with access to all relevant information including the electronic health record system of St. Olav`s University Hospital (Helseplattformen, EPIC Systems Corporation), live streaming from prehospital patient monitors (CorPuls Mission; GS Elektromedizinische Geräte G. Stemple GmbH, Kaufering, Germany) and live on-scene video transmission (“Hjelp 113 Video”, Norwegian Air Ambulance Foundation, Oslo, Norway) [26].

EMCC operators and participating physicians were given a detailed task instruction on how to interact during the intervention. Whenever the EMCC operator received an emergency call where HEMS dispatch was either indicated according to current dispatch criteria, or the operator wanted to consult a physician regarding a potential HEMS dispatch, the on-site EP should be contacted without delay to make the final decision on whether or not to dispatch HEMS. If the HEMS crew was tasked, the HEMS physician on-call was instructed to accept the mission without further medical questioning. However, the HEMS physician could override the on-site EP`s decision and reject a mission if major operative concerns were identified. Operational flight considerations including weather assessments were done by the HEMS pilot as normal.

The on-site EPs were generally instructed to obtain a passive role, i.e. “speak when spoken to” during shifts in the EMCC. Operators were free to contact the EP whenever they felt it was necessary, except in HEMS dispatch considerations where consulting was required. The EPs were further instructed to silently listen to all 113-calls with acute triage to be able to respond quickly upon request for assistance by the operator. If the EP chose to actively involve oneself in an emergency call without being asked, this should be documented specifically.

### Study design

In this quasi-experimental study, the intervention with an on-site EP was introduced exclusively in the intervention group and not randomly assigned. This intervention group (denoted A2 in Table 1) was defined as all working hours with an on-site EP in the EMCC. We also included control groups covering the corresponding days and time intervals for both working and non-working hours in 2022 and 2023 (groups A1 and B1), as well as non-working hours during the intervention period (group B2, Table 1. Participating physicians and EMCC operators were blinded for the endpoints of the study.

**Table 1:** Group design for working and non-working hours

	Pre-intervention period Feb 1 to Jul 5 years 2022 and 2023	Intervention period Feb 1 to Jul 5 year 2024
**Working hours** Mon-Thu 10am-10pm Fri 8am to 4pm	No intervention (A1)	Intervention (A2)
**Non-working hours** Mon-Thu 10pm-10am Fri 4pm-Mon 10am	No intervention (B1)	No intervention (B2)

### Primary outcome

The primary outcome of the study was *unnecessary HEMS dispatch*, defined as a mission where neither *advanced treatment* nor *logistical contributions* were provided by dispatching HEMS to the scene. This was measured from a panel of quality indicators (QI) for physician-staffed emergency medical services that has been registered by the HEMS physician on every dispatch at Trondheim HEMS since September 7th 2021 [27]. If the response to both QIs was “No” following a HEMS mission, this was registered as an unnecessary HEMS dispatch (Table 2).

**Table 2:** Definition of quality indicators for primary outcome

Quality indicator	Advanced treatment	Logistics
	*Did the HEMS crew provide advanced treatment in the actual response?*	*Did the logistical contribution by HEMS give the patient a significant better service than the existing alternative?*
Response	1. Yes; procedures (both medical and rescue techniques) or medications only offered by P-EMS units in the actual region.	1. Yes; by reducing the estimated time to admitting facility with ≥ 30 min for time critical conditions like STEMI, stroke and severe trauma.
	2. Yes; procedures or medications also offered by other local pre-hospital units than the P-EMS, but these were not present on scene.	2. Yes; by reducing the estimated time to admitting facility with 15–29 min for time critical conditions like STEMI, stroke and severe trauma.
	3. Yes; avoidance of unethical/unnecessary treatment	3. Yes; by accessing and/or evacuating the patient from an area otherwise difficult to access.
	4. Yes; presence in particularly demanding situations, e.g. the death of a child, major incidents etc. (The presence of P-EMS was considered supportive for both other caregivers and relatives).	4. Both 1 and 3. 5. Both 2 and 3.
	5. No	6. No

HEMS: Helicopter Emergency Medical Services, P-EMS: Physician-staffed Emergency Medical Services, STEMI: ST-Elevation Myocardial Infarction

### Secondary outcomes

Secondary outcomes were HEMS alarm time (time from emergency call to HEMS alarm), HEMS activation time (time from HEMS alarm to HEMS take-off), rejected HEMS missions and National Advisory Committee for Aeronautics (NACA)-scores of encountered HEMS patients being 4 or higher. In the eight-level NACA scoring system, the most serious clinical state during a mission is registered, where NACA 0 indicates no injury or illness and NACA 7 means that the patient is declared dead during the mission (with or without resuscitation attempts) [28].

### Inclusion and exclusion criteria

**Fig. 1:**
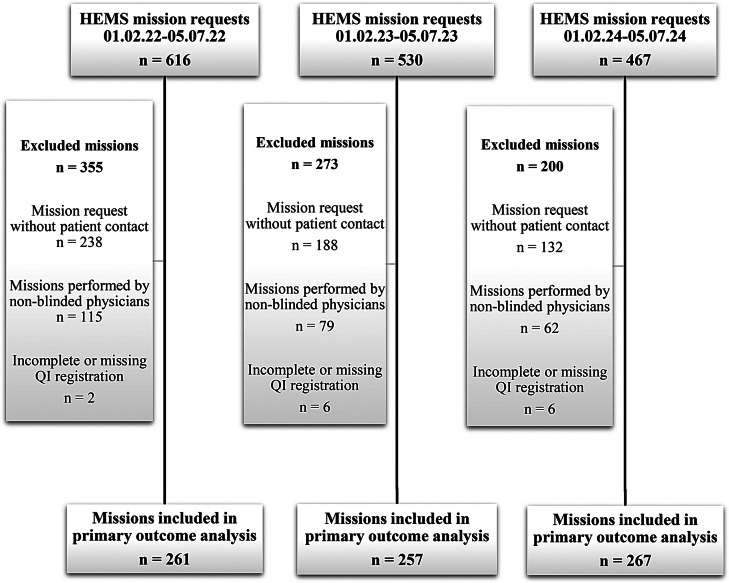
Flow chart for inclusion and exclusion for primaryoutcome analysis. HEMS: Helicopter Emergency Medical Services, QI: Quality Indicator

All requests to Trondheim EMCC regarding potential missions for Trondheim HEMS during the pre-intervention and intervention periods, either assessed by the on-site EP (Table 1; group A2) or by the HEMS physician (Table 1; group A1, B1 and B2), were included in the study. Missions without patient contact were excluded from the analyses as quality indicators were not registered in these cases. Also, missions performed by two HEMS physicians in the study group (Haugland and Uleberg) who were not blinded for outcome measures and missions with incomplete or missing QI registration were excluded (Fig. 1).

### Sample size and statistical analysis

Historical data showed that the proportion of unnecessary HEMS dispatches without medical or logistical benefit were 19% in both 2022 and 2023, respectively. An estimated effect size of 50% reduction of unnecessary dispatches (to 9.5%) was considered appropriate. Based on 80% power, an α-level of 0.05 and a κ-level of 2, the required sample size was found to be 142 missions with patient contact [29]. Based on 849 missions with patient contact in 2022, this implied an intervention period of approximately 5 months to reach the required sample size. The final intervention period was February 1st through July 5th, 2024.

To assess the association between the intervention and selected outcomes we performed a difference-in-differences (DiD) analysis, which is a frequently used method for evaluating the impact of non-randomized interventions in healthcare [30–32]. A DiD design compares changes over time in an intervention group with changes in a group that does not receive the intervention, often summarised in a 2 × 2 table as presented in Table 1. In the pre-intervention period, groups A1 and B1 were “exposed” to the control condition without an on-site EP. In the intervention period, group A2 was exposed to the intervention with an on-site EP while the control group B2 was unexposed. The DiD estimate, which reflects the intervention effect, was calculated as (A2-A1) - (B2-B1). We estimated the DiD with ordinary linear regression with an interaction term between working-time and intervention year, providing estimates of risk differences for binary outcomes. In addition, we analysed the data by comparing working hours in the intervention period with all other periods where no intervention occurred (groups A2 vs. A1, B2 and B1). For this analysis we used logistic and ordinary linear regression for binary and continuous outcomes, respectively, adjusted for factors that could influence differences over time; day of the year (linear), time of the day (five-point spline) and age and sex of the patient. All analyses were adjusted for the on-site EP-identifier to account for possible different practices among different EPs.

Epidemiological data were analysed by Student’s t-test or Pearson’s χ2 test, as appropriate. Data are reported as mean with SD, median with IQR or proportions, as appropriate. Statistical analyses were performed using IBM Statistics SPSS 29 (IBM Corp. Released 2020. IBM SPSS Statistics for Windows, Version 27.0. Armonk, NY: IBM Corp), R Statistics 4.0.4 (R Core Team 2013, R Foundation for Statistical Computing, Vienna, Austria) and Microsoft Excel (Microsoft Office 365 ProPlus, Microsoft Corporation, USA). Results are reported with a significance level of 0.05.

### Data sources, collecting and cleaning

Routinely collected data on all HEMS requests were gathered retrospectively from the Trondheim EMCC database AMIS and the HEMS database LABAS. *AMIS* is the emergency medicine information system providing EMS data including patient status, ambulance dispatch and timeline data. *LABAS* (Normann IT, Trondheim, Norway) is the operational database and medical record generator of the Norwegian HEMS service. Quality indicators were registered electronically after each HEMS mission by the HEMS physician on-call and collected retrospectively. Data were stored on a secure server at Central Norway Regional Health Authority`s IT department (HEMIT).

For each HEMS request we registered timeline parameters (time of emergency call, HEMS alarm, HEMS take-off), patient characteristics (age, sex, International Classification of Diseases [ICD] -10 diagnosis, NACA severity score), HEMS mission data (type of mission, mission triage, transportation by car or helicopter, deviations (rejected or aborted missions, cause of deviations) and selected quality indicators (advanced treatment or logistical contribution provided by the HEMS crew).

During working hours in the intervention period, the on-site EP and EMCC operator were instructed to complete a questionnaire every time the EP was involved in an emergency call. The questionnaire included reasons for and consequences of involving the EP, deviations from EMCC routines and a usefulness score for the EP involvement. At the same time, the HEMS physician on-call completed a questionnaire for all HEMS requests, including usefulness scores for information provided by the on-site EP in different stages of the HEMS missions. Data were registered on paper continuously during EMCC and HEMS shifts and transferred electronically to a web-based database (eFORSK, HEMIT). The complete questionnaires are presented in Additional file 3.

The complete dataset was assessed by the main author prior to analyses to identify multiple registrations of a single event for both LABAS and AMIS data. If two or more patients were encountered on a single HEMS mission, only the patient with the highest NACA score was included in the primaryendpointanalysis. HEMS missions with patient contact that had incomplete registration of quality indicators (*n* = 268) were sent to the individual HEMS physicians without unblinding for post-registration as part of a general data cleaning process at the Trondheim HEMS base.

## Results

In total, an on-site EP was present at Trondheim EMCC during 960 work hours distributed over 100 weekdays throughout the intervention period. In total, 8543 emergency calls were responded to by the EMCC operators with an on-site EP (group A2).

Trondheim HEMS base received 1146 and 467 HEMS mission requests during the pre-intervention and intervention period, respectively. When excluding missions without patient contact, missions executed by the two HEMS physicians in the study group and missions with incomplete or missing quality indicator registration, 518 (pre-intervention period) and 267 (intervention period) HEMS missions were included in the primary outcome analysis (Fig. 1).

Baseline data for HEMS missions during working and non-working hours are presented in Table 3. There were no significant differences between non-working and working hours in the pre-intervention and intervention periods regarding patient age, gender and ICD-10 diagnoses (Additional file 1).

### Primary outcome

In 33 (working hours) and 30 (non-working hours) percent of HEMS missions during the intervention period, respectively, the HEMS physician judged the mission unnecessary, i.e. that no advanced treatment or logistical contribution was provided (Table 3). There was no significant difference in the risk of an unnecessary mission when comparing working and non-working hours in the pre-intervention and intervention periods by difference-in-difference analysis (risk difference [RD] 5.6, 95% confidence interval [CI] -7.4–18.6) (Table 4). Comparing working hours in the intervention period with all controls from 2022 to 2024 combined gave consistent results (odds ratio [OR] 1.36], 95% CI 0.82–2.26) (Table 4).

**Table 3:** Outcome and baseline data for HEMS missions in pre-intervention and intervention periods

	February to July, 2022 and 2023		February to July, 2024		
	Non-working hours	Working hours	Non-working hours	Working hours (intervention)	Overall
	*N* (%)	*N* (%)	*N* (%)	*N* (%)	*N* (%)
HEMS missions with patient contact	296 (100)	222 (100)	169 (100)	**98 (100)**	785 (100)
Outcomes					
No advanced treatment	172 (58)	131 (59)	105 (62)	**67 (68)**	475 (61)
No logistical contribution	141 (48)	94 (42)	91 (54)	**50 (51)**	376 (48)
Unnecessary mission	76 (26)	46 (21)	51 (30)	**32 (33)**	205 (26)
Median time, call to alarm [IQR]	6 [25]	5 [19]	7 [17]	**7 [15]**	6 [19]
Patient characteristics					
Mean age [SD]	51 [27]	54 [27]	55 [24]	**51 [26]**	53 [26]
Women	105 (35)	87 (39)	54 (32)	**30 (31)**	276 (35)
NACA score from 4 to 7	230 (78)	180 (81)	138 (82)	**75 (77)**	623 (79)
ICD10, chapter I	116 (39)	98 (44)	80 (47)	**39 (40)**	333 (42)
ICD10, chapter S	48 (16)	34 (15)	19 (11)	**22 (22)**	123 (16)
ICD10, chapter R	32 (11)	31 (14)	23 (14)	**15 (15)**	101 (13)
ICD10, chapter J	30 (10)	14 (6)	11 (7)	**5 (5)**	60 (8)

HEMS: Helicopter emergency services, IQR: Interquartile range, SD: standard deviation

**Table 4:** Primary and secondary outcome analyses

Outcome	Unit	Difference in differences, Estimate (95% CI)^1^	Unit	Intervention versus non-intervention, Estimate (95% CI)^1,2^
Unnecessary mission	%-point RD	5.6 (-7.4 to 18.6)	OR	1.36 (0.82 to 2.26)
NACA score 4 to 7	%-point RD	-5.8 (-17.9 to 6.3)	OR	1.20 (0.69 to 2.11)
Time from 113 call to HEMS alarm	Minutes	0.9 (-7.7 to 9.5)	Minutes	0.7 (-5.6 to 7.0)
Time from HEMS alarm to take-off	Minutes	3.1 (-1.9 to 8.1)	Minutes	2.1 (-1.5 to 5.7)

^1^Analyses were adjusted for EMCC physician. ^2^Analyses were adjusted for day of the year, time of the day and age and sex of the patient. OR: Odds ratio, CI: Confidence interval, RD: Risk difference, NACA: National Advisory Committee for Aeronautics

### Secondary outcomes

Overall, 79% of all HEMS missions during working and non-working hours included patients with a high severity score (NACA score of 4–7) (Table 3). We did not demonstrate a significant difference in the proportion of HEMS missions assessing patients with NACA scores of 4 or higher when comparing intervention and non-intervention groups, including the difference-in-difference analysis (RD -5.8, 95% CI -17.9–6.3) (Table 4).

The overall median time from emergency call to HEMS alarm was 6 min in the dataset (Table 3). A significant change in HEMS alarm time, or time from alarm to mission start (HEMS take-off) was not demonstrated after the intervention (Table 4).

Table 5 presents a comparison of the proportion of HEMS mission requests rejected due to lack of medical indication for each individual physician in the role as HEMS physician (group A1, B1 and B2, Table 1) and on-site EP (group A2, Table 1), respectively. All participating physicians rejected a higher proportion of mission requests individually when being the on-site EP, compared to their decisions during HEMS shifts (3.8–25.5% point increase). For all seven physicians together, this on-site EP rejection rate was significantly higher (12.6% point increase, *p* < 0.001) (Table 5).

**Table 5:** Individual HEMS mission rejections as HEMS physician and on-site EP

Physician	Rejected/requests as HP (%)	Rejected/requests as EP (%)	Difference (%-points)	*P*-value
A	9/122 (7.4)	10/34 (29.4)	22.0	
B	5/119 (4.2)	2/11 (18.2)	14.0	
C	25/255 (9.8)	9/59 (15.3)	5.5	
D	36/179 (20.1)	14/31 (45.2)	25.5	
E	12/253 (4.7)	9/39 (23.1)	18.3	
F	4/203 (2.0)	3/29 (10.3)	8.4	
G	9/154 (5.8)	5/52 (9.6)	3.8	
A-G	100/1285 (7.8)	52/255 (20.4)	12.6	< 0.001^1^

HEMS: Helicopter Emergency Medical Services, HP: HEMS hysician, EP: Emergency Medical Communication Centre Physician. ^1^Pearson Chi-square test

### Response to questionnaires

In total, 983 questionnaires were completed by the on-site EPs (*n* = 519), EMCC operators (*n* = 301) and HEMS physician (*n* = 163) on 563 emergency calls during the intervention period. This implies that the on-site EP on average was involved in 5.6 cases each shift, equivalent to one consultation every 1.7 h. Of all 8543 emergency calls responded to during working hours in the intervention period, involvement of the on-site EP was documented in 6.6% of these calls.

In 180 of the 563 calls involving an on-site EP, the EP took the initiative to get involved without any request from the EMCC operator. In these cases, the EMCC operator response rate was low (*n* = 37). This implies a response rate of 92% (519/563) for EPs, and 69% (264/383) for the EMCC operators in cases where the operator contacted the EP. Trondheim HEMS received 180 mission requests during working hours in the intervention period, with a corresponding HEMS physician response rate of 91% (163/180).

The on-site EP was contacted by an EMCC operator or a HEMS coordinator in 39.5% and 20.8% of all cases involving an EP, respectively. Personnel from ground ambulances contacted the EP in only 1.3% of cases, while the EP got involved by own initiative in 36.6% of all cases. To clarify the EMCC operators` intentional use of an on-site EP, cases where the EP was involved by own initiative only were excluded from the response to questionnaires presented in Additional file 2.

## Discussion

In this quasi-experimental study of HEMS dispatches in a Norwegian EMCC we investigated transferring the medical dispatch decision from the HEMS physician to an on-site physician in the EMCC. We found no evidence of increased HEMS dispatch precision, measured by the proportion of HEMS missions where no advanced treatment or logistical contribution was provided following the intervention. A minor increase in such unnecessary HEMS missions was observed after the intervention, but the results were compatible with no statistically significant effect. Furthermore, we found no significant change in HEMS missions with severely ill or injured patients (NACA-scores 4–7).

Various studies have addressed the challenges of appropriate HEMS dispatch [1, 2, 33–36]. General HEMS dispatch criteria have proven difficult to establish, and the final decision on whether or not to dispatch a helicopter might require a complex decision process relying on the experience of EMCC operators and HEMS crew [1]. Bringing an experienced clinician closer to the decision-making in the EMCC may provide a more appropriate dispatch [19]. To our knowledge, this is the first study to evaluate the effect on HEMS dispatch from bringing a HEMS physician into the EMCC.

A major shift in the decision process for HEMS dispatches was introduced in this study. Interventions to improve quality in healthcare might face several barriers including staff engagement, consistent data collection and protocol adherence [37]. HEMS physicians in this study reported that HEMS dispatch was decided by the on-site EP alone in nearly 90% of all events, indicating good adherence to the study protocol. There were only a few cases where the HEMS physicians rejected the request despite the on-site EPs decision (*n* = 2, 1.2% of all HEMS physician questionnaires). Involving the on-site EP led to minor deviations from standard operating procedures in the EMCC in only 1.9% of all cases, and, according to the HEMS crew, involvement of the on-site EP did not affect the mission performance negatively in 93% of the events. These findings indicate that the intervention was both safe and feasible. We found no evidence to indicate that involving an on-site EP in HEMS dispatch caused delays in the time from an emergency 113 call to HEMS alarm or from HEMS alarm to mission start. However, while the differences were not statistically significant, we did observe slightly longer timelines when an on-site EP was present (Table 4). As HEMS activation time has been shown to be influenced by the number of intermediators involved in emergency calls, this observation is noteworthy [38].

An argument for a present physician in the EMCC has been the immediate availability of a dedicated experienced consultant without other concurrent operative duties [10]. In our study, EMCC operators responded that the on-site EP was available within two minutes in 97% of cases. Despite the high availability, the EP was involved in only 6.6% of all emergency calls during working hours in the intervention period. The passive role of the EP might partly explain the low degree of EP involvement. However, this strategy was deliberately chosen to standardize the actions of the seven individual EPs in an unfamiliar setting in the EMCC. Although operators rated the usefulness of EP involvement as quite high, they responded that they would not have contacted the EP if not present in the EMCC in nearly 60% of all cases. While the educational level of Emergency Medical Dispatchers (EMD) varies notably between different EMCC systems internationally, our findings might reflect that Norwegian EMCC operators, all medically educated as either Emergency Medical Technicians (EMT), nurses or paramedics, are used to operate autonomously supported by current dispatch guidelines without consulting an EP [21, 39–41].

Various endpoints have been assessed to describe HEMS dispatch precision in different EMCC systems, including the eight-level National Advisory Committee for Aeronautics (NACA) score which is well-established in prehospital emergency services of Western Europe [17]. As capturing all aspects of a complex dispatch process by a single scoring system is challenging, a multidimensional approach based on medical and logistical contribution in a HEMS mission may be valuable [27, 42]. The proportion of encountered HEMS patients with a NACA score of 4 or higher, indicating severe illness or injury, was 78–82% in all groups in our data. These numbers are higher than previously reported results from comparable services, indicating proper HEMS dispatch [43, 44]. However, the HEMS physicians in our study reported that no medical contributions were provided in 58–68% of all missions, which is somewhat higher than previous data from Nordic HEMS services using the same quality indicators [45]. Given the wide definition of the medical contribution in the “advanced treatment” quality indicator, including non-technical skills like care for patients` relatives in special clinical circumstances and withdrawal of unethical treatment, this high proportion of medically unnecessary HEMS missions observed in our data is noteworthy. Although clearly defined, different interpretations and documentation practices of the quality indicators amongst HEMS physicians could possibly explain this finding.

The HEMS dispatch decision involves multiple contextual factors in addition to medical details of the patients involved, including available ground resources, distance to receiving hospital and available information at the time of dispatch. In a complex decision process with limited available time, individual differences in personality and experience between HEMS physicians will likely affect the decision making process [46]. A possible explanation for the generally increased mission rejection rate during working hours might be a lower threshold for EMCC operators to discuss HEMS dispatch with an on-site EP compared to a more remotely located HEMS physician on-call. Comparing individual HEMS physicians, we found considerable variations in the proportion of missions being rejected due to a perceived lack of medical indication (a range of 2–20%). Interestingly, we observed an even greater variability when comparing the proportion of rejected missions for each individual in the role as on-site EPs (10–45%). As our data does not support an improvement in HEMS dispatch precision by moving the HEMS physician into the EMCC, finding ways to reduce variation in mission acceptance between different HEMS physicians could be a path for future studies regarding HEMS resource utilization.

### Strengths and limitations

All participating operators and five out of seven participating physicians were blinded for the study outcomes. A randomized controlled study design was considered infeasible, which limits robust causality conclusions following the intervention. However, a difference-in-difference analysis was included to address limitations with before-after-studies, including confounding due to unmeasured temporal changes and other unidentified differences between intervention and control groups. Also, the subjective nature of scoring quality indicators for participating HEMS physicians is a limitation in assessing the primary outcome. Due to substantial costs related to introducing a present EP, an ambitious effect size estimate for the primary outcome was chosen. This implies that the study was underpowered to assess the significance of smaller observed changes in the selected outcomes. Different models for HEMS dispatch including EMCC staffing and organization may challenge the external validity of the study.

## Conclusion

In this quasi-experimental study in a Norwegian EMCC and HEMS base, the medical decision regarding HEMS dispatch was transferred from the HEMS physician on-call to an on-site EMCC physician. We found no support for an increase in HEMS dispatch precision measured by the proportion of missions without medical or logistical contributions following the intervention.

## Electronic supplementary material

Below is the link to the electronic supplementary material.

Supplementary Material 1: Additional file 1 (PDF): Baseline data for HEMS patients in intervention and non-intervention periods.

Supplementary Material 2: Additional file 2 (PDF): Responses to questionnaires.

Supplementary Material 3: Additional file 3 (PDF): Questionnaires.

## Data Availability

Data are available upon reasonable request.

## Abbreviations

DiD: Difference-In-Differences
EMCC: Emergency Medical Communication Centre
EMS: Emergency Medical Services
EMT: Emergency Medical Technician
EP: EMCC physician
GP: General Practitioner
HEMS: Helicopter Emergency Medical Service
HP: HEMS physician
