# Supplementary material for: Introducing an on-site Helicopter Emergency Medical Service (HEMS) physician at the Emergency Medical Communication Centre - implications for dispatch precision at a Norwegian HEMS base
Source: Scand J Trauma Resusc Emerg Med. 2025 May 7;33:80. doi: 10.1186/s13049-025-01396-1 (PMC12057114; doi:10.1186/s13049-025-01396-1)
Supplement: Supplementary file 1 — Supplementary Material 1: Additional file 1 (PDF): Baseline data for HEMS patients in intervention and non-intervention periods. [file 13049_2025_1396_MOESM1_ESM.pdf]

Additional file 1. Baseline data for HEMS patients in intervention and non-intervention periods

Factor	Unit	Intervention versus non-intervention, Estimate (95% CI)
Age	Years	-3.2 (-8.7 to 2.3)
Woman	%-point RD	-5.5 (-15.5 to 4.6)
ICD-10, chapter I	%-point RD	-3.3 (-13.8 to 7.1)
ICD-10, chapter J	%-point RD	-1.1 (-6.8 to 4.5)
ICD-10, chapter S	%-point RD	7.7 (0.1 to 15.4)
ICD-10, chapter R	%-point RD	2.3 (-4.7 to 9.4)

ICD: International Classification of Diseases, RD: Risk difference
