# Supplementary material for: Introducing an on-site Helicopter Emergency Medical Service (HEMS) physician at the Emergency Medical Communication Centre - implications for dispatch precision at a Norwegian HEMS base
Source: Scand J Trauma Resusc Emerg Med. 2025 May 7;33:80. doi: 10.1186/s13049-025-01396-1 (PMC12057114; doi:10.1186/s13049-025-01396-1)
Supplement: Supplementary file 2 — Supplementary Material 2: Additional file 2 (PDF): Responses to questionnaires. [file 13049_2025_1396_MOESM2_ESM.pdf]

Additional file 2. Responses to questionnaires

	EP		EMCC operator		HEMS physician	
	N (n) *Mean* (SD)	%	N (n) *Mean* (SD)	%	N (n) *Mean* (SD)	%
Why was the EP contacted?*	338(1)		264			
Decision support for HEMS dispatch	217	64.2	141	53.4		
Medical advice in emergency calls	58	17.2	34	12.9		
Decision support logistics	57	16.9	52	19.7		
Decision support triage level	65	19.2	47	17.8		
How available was the EP upon requests?	339		264			
Immediately available	315	92.9	236	89.4		
Available within 2 minutes	18	5.3	19	7.2		
Available within 5 minutes	2	0.6	6	2.3		
Not available within 5 minutes	4	1.2	3	1.1		
*If the EP was contacted for HEMS dispatch decision support, what were the consequences of involving the EP *?*	217					
Trondheim HEMS alarmed	117	53.9				
Another HEMS base alarmed	31	14.3				
HEMS alarm rejected	47	21.7				
Additional information collected	47	21.7				
Change in prehospital resource allocation	13	6.0				
If the physician wasn`t physically present in the EMCC, would you still have contacted the EP in this case?			259(5)			
Yes			111	42.9		
No			148	57.1		
Did involvement of the EP lead to deviations from standard operating procedures in the EMCC?	337(2)		260(4)			
No	301	89.3	239	91.9		
Yes	5	1.5	5	1.9		
Uncertain	31	9.2	16	6.2		
On a scale from 1 (no utility value) to 5 (major utility value), how useful was the overall EP contribution in this event?	339		260		154	
Mean score (SD)	*3.41* (0.9)		*3.72*(1.1)		*2.33*(1.2)	
T-test of difference in means (p-value): EP vs EO	*- 0.32* (<0.01)					
T-test of difference in means (p-value): EP vs HP	*1.1* (<0.01)					
Was the HEMS physician involved in the decision regarding HEMS alarm?	133				163	

□ HEMS alarm was decided by EP alone	107	80.5			141	86.5
□ HEMS alarm was decided by EP in accordance with the HP	19	14.3			14	8.6
□ EP decided HEMS alarm, but HP rejected the request	1	0.8			2	1.2
□ Mission not completed due to operative reasons	6	4.5			3	1.8
Why did the HP reject the mission despite the EP`s decision to alarm the HEMS crew?					18	
□ Lack of medical indication for HEMS activation					1	5.6
□ Cost/benefit considerations within the HEMS crew					2	11.1
□ Concurrencies					1	5.6
□ Weather conditions					2	11.1
□ Other reasons					5	27.8
□ HP rejected the mission without involvement of the EP					9	50
On a scale from 1 (obvious lack of information) to 5 (clearly sufficient information), to what degree was the HEMS crew sufficiently informed at the time of HEMS alarm?					163	
Mean (SD)					*3.59* (0.9)	
On a scale from 1 (no utility value) to 5 (major utility value), how useful was the EP contribution during the HEMS mission?						
□ Information at the time of HEMS alarm					155<br>*2.37* (1.3)	
□ Information during flight to patient					152<br>*2.35* (1.2)	
□ Medical advice to on-scene personnel					139<br>*1.83* (1.0)	
□ Information to the receiving hospital prior to HEMS arrival					134<br>*1.75* (1.0)	
According to the HEMS crew, did the involvement of the EP affect the mission performance negatively?					157	
□ The mission performance was not negatively affected					144	92.9
□ Mission logistics were affected negatively					5	3.2
□ Medical planning of the mission was affected negatively					2	1.3
□ Situational awareness was affected negatively					1	0.6
□ Other negative consequences of EP involvement					3	1.9

* Multiple response question, (n) missing values, EP: EMCC physician, EO: EMCC operator, HP: HEMS physician
