# Supplementary material for: Introducing an on-site Helicopter Emergency Medical Service (HEMS) physician at the Emergency Medical Communication Centre - implications for dispatch precision at a Norwegian HEMS base
Source: Scand J Trauma Resusc Emerg Med. 2025 May 7;33:80. doi: 10.1186/s13049-025-01396-1 (PMC12057114; doi:10.1186/s13049-025-01396-1)
Supplement: Supplementary file 3 — Supplementary Material 3: Additional file 3 (PDF): Questionnaires. [file 13049_2025_1396_MOESM3_ESM.pdf]

Questionnaires

EMCC physician

AMIS number: ______________________

Time of emergency call (dd/mm/hh:mm): __________________

Time of contact with EMCC physician: (dd/mm/hh:mm): __________________

Choose one or more alternatives

1. **Who contacted the EMCC physician?**
   - ☐ Medical EMCC operator
   - ☐ EMCC resource coordinator
   - ☐ HEMS coordinator
   - ☐ Ground ambulance personnel
   - ☐ EMCC physician got involved in the case by own initiative only
   - ☐ Others: _______________

2. **Why was the EMCC physician contacted?**
   - ☐ Decision support for triage level
   - ☐ Medical advice in emergency calls
   - ☐ Decision support for HEMS dispatch
   - ☐ Decision support logistics
   - ☐ Concurrencies
   - ☐ Other reasons: ____________________

3. **How available was the EMCC physician upon request?**
   - ☐ Immediately available
   - ☐ Available within 2 minutes
   - ☐ Available within 5 minutes
   - ☐ Not available within 5 minutes

4. What were the consequences of involving the EMCC physician in this case?

- ☐ Trondheim HEMS dispatched
- ☐ Dispatch of Trondheim HEMS considered not necessary
- ☐ Dispatch of other HEMS resources in the region
- ☐ Downscaling of triage level
- ☐ Upscaling of triage level

- ☐ Adjusted medical advice to caller or ground personnel on-site
- ☐ Acquisition of additional information from caller, local resources or medical health records
- ☐ Adjusted dispatch of prehospital resources
- ☐ Adjusted patient destination: dispatch of ground ambulance, but patient was left home or at an institution
- ☐ Adjusted patient destination: admission to out-of-hours medical services instead of hospital
- ☐ Emergency department team activation (trauma team or other)
- ☐ Emergency department team cancellation (trauma team or other)
- ☐ Other consequences: _____________
- ☐ No consequences

5. If the EMCC physician involvement resulted in HEMS dispatch: Was the HEMS physician also involved in the dispatch decision?

- ☐ HEMS dispatch was decided by the EMCC physician alone
- ☐ HEMS dispatch was decided by the EMCC physician in accordance with the HEMS physician
- ☐ EMCC physician decided HEMS dispatch, but the HEMS physician rejected the request
- ☐ EMCC physician decided HEMS dispatch, but the mission was not completed due to operative reasons
- ☐ Not applicable

6. If the EMCC physician was contacted due to HEMS dispatch considerations, why was HEMS dispatch rejected?

- ☐ The mission did not involve a time-critical condition
- ☐ There was no need for advanced medical competence on-site
- ☐ There was no significant logistical contribution by dispatching HEMS to the scene
- ☐ Other: ___________
- ☐ Not applicable

7. Did involvement of the EMCC physician lead to deviations from standard operating procedures in the EMCC?

- ☐ No
- ☐ Yes
- ☐ Unsure

8. In your opinion, how useful was the overall contribution of the EMCC physician in this case?

1 no utility value – 5 major utility value

☐ 1 ☐ 2 ☐ 3 ☐ 4 ☐ 5

☐ **EMCC operator** ☐ **HEMS coordinator**

AMIS number: ______________________

Time of emergency call (dd/mm/hh:mm): __________________

Time of contact with EMCC physician: (dd/mm/hh:mm): __________________

Choose one or more alternatives

1. Why was the EMCC physician contacted?

- ☐ Decision support triage level
- ☐ Medical advice in emergency calls
- ☐ Decision support for HEMS dispatch
- ☐ Decision support logistics
- ☐ Concurrencies
- ☐ Other reasons: ____________________
- ☐ EMCC physician got involved by own initiative only

2. How available was the EP upon request?

- ☐ Immediately available
- ☐ Available within 2 minutes
- ☐ Available within 5 minutes
- ☐ Not available within 5 minutes

4. What were the consequences of involving the EMCC physician in this case?

- ☐ Trondheim HEMS dispatched
- ☐ Dispatch of Trondheim HEMS considered not necessary
- ☐ Dispatch of other HEMS resources in the region
- ☐ Downscaling of triage level
- ☐ Upscaling of triage level
- ☐ Adjusted medical advice to caller or ground personnel on-site
- ☐ Acquisition of additional information from caller, local resources or medical health records
- ☐ Adjusted dispatch of prehospital resources
- ☐ Adjusted patient destination: dispatch of ground ambulance, but the patient was left home or at an institution
- ☐ Adjusted patient destination: admission to out-of-hours medical services instead of hospital
- ☐ Emergency department team activation (trauma team or other)
- ☐ Emergency department team cancellation (trauma team or other)

- ☐ Other consequences: ______________
- ☐ No consequences

5. **Did involvement of the EMCC physician lead to deviations from standard operating procedures in the EMCC?**
   - ☐ No
   - ☐ Yes
   - ☐ Unsure

6. **In your opinion, how useful was the overall contribution of the EMCC physician in this case?**
   1 no utility value – 5 major utility value
   1 ☐ 2 ☐ 3 ☐ 4 ☐ 5

7. **If the EMCC physician wasn`t physically present in the EMCC, would you still have contacted the physician in this case?**
   - ☐ Yes
   - ☐ No

HEMS physician

AMIS number: ______________________

Time of emergency call (dd/mm/hh:mm): __________________

Time of contact with EMCC physician (dd/mm/hh:mm): __________________

Choose one or more alternatives

1. **Were you as the HEMS physician on-call involved in the HEMS dispatch decision?**
   - ☐ HEMS dispatch was decided by the EMCC physician alone
   - ☐ HEMS dispatch was decided by the EMCC physician in accordance with the HEMS physician
   - ☐ EMCC physician decided HEMS dispatch, but the HEMS physician rejected the request
   - ☐ EMCC physician decided HEMS dispatch, but the mission was not completed due to operative reasons
   - ☐ Not applicable

2. **If Trondheim HEMS rejected HEMS dispatch despite the EMCC physician`s decision, what were the reasons for this rejection?**
   - ☐ Lack of medical indication for HEMS dispatch at time of alarm
   - ☐ An overall risk-benefit assessment of HEMS dispatch in the actual case within the HEMS crew
   - ☐ Technical status of the helicopter
   - ☐ Concurrencies
   - ☐ Weather conditions
   - ☐ Duty time regulations
   - ☐ Other reasons: ______________
   - ☐ Not applicable

3. **To what degree was the HEMS crew sufficiently informed at the time of HEMS alarm?**

   1 obvious lack of information - 5 clearly sufficient information

   1 ☐ 2 ☐ 3 ☐ 4 ☐ 5

4. In your opinion, how useful was the contribution of the EMCC physician in this case?

☐ Not applicable (EMCC physician not involved in the case)

Information at the time of HEMS alarm

1 no utility value – 5 major utility value

1 ☐ 2 ☐ 3 ☐ 4 ☐ 5

Information en route to patient

1 no utility value – 5 major utility value

1 ☐ 2 ☐ 3 ☐ 4 ☐ 5

Medical treatment and guidance to on-scene resources before HEMS arrival

1 no utility value – 5 major utility value

1 ☐ 2 ☐ 3 ☐ 4 ☐ 5

Information to receiving hospital before patient admission

1 no utility value – 5 major utility value

1 ☐ 2 ☐ 3 ☐ 4 ☐ 5

How useful was the overall contribution of the EMCC physician in this case?

1 no utility value – 5 major utility value

☐ 1 ☐ 2 ☐ 3 ☐ 4 ☐ 5

5. According to the HEMS crew, did the involvement of the EMCC physician affect the mission performance negatively?

☐ The mission performance was not negatively affected
☐ Mission logistics were affected negatively
☐ Medical planning of the mission was affected negatively
☐ Situational awareness was affected negatively
☐ Other negative consequences of EP involvement
☐ Not applicable
